# Nerofe+ldDox releases c-Jun from nuclear ST2 to reprogram the immune microenvironment in mtKRAS tumors

**DOI:** 10.18632/oncotarget.28820

**Published:** 2025-12-24

**Authors:** Joel Ohana, Uziel Sandler, Benjamin A. Weinberg, Stephen Liu, Yoram Devary

**Affiliations:** ^1^Immune System Key (ISK) Ltd., Jerusalem 9746009, Israel; ^2^Department of Bio-Informatics, Lev Academic Center (JCT), Jerusalem 91160, Israel; ^3^Ruesch Center for the Cure of Gastrointestinal Cancers, Lombardi Comprehensive Cancer Center, Georgetown University, Washington, DC 20007, USA

**Keywords:** mutant KRAS, Nerofe, ST2 Receptor, tumor immune microenvironment, nuclear immunomodulation

## Abstract

Background/Objectives: Mutant KRAS (mtKRAS) tumors are highly immunosuppressive, largely through secretion of IL-10 and TGF-β2, which prevent immune cell infiltration. Nerofe (dTCApFs), a peptide derivative of Tumor Cell Apoptosis Factor, induces endoplasmic reticulum stress and modulates immune signaling through the T1/ST2 receptor, which is overexpressed in mtKRAS tumors. We evaluated whether combining Nerofe with low-dose doxorubicin (ldDox) could remodel the immune microenvironment and overcome tumor immunosuppression.

Methods: *In vitro* experiments were performed in PANC-1 pancreatic adenocarcinoma cells harboring a KRAS mutation. Cytokine expression, c-Jun activity, and c-Jun–ST2 binding were measured by western blotting, immunocytochemistry, and immunoprecipitation. In a clinical trial (NCT05661201), patients with mtKRAS tumors received weekly Nerofe (288 mg/m²) plus ldDox (8 mg/m²). Tumor biopsies were analyzed by immunohistochemistry before treatment and after 7 weeks.

Results: Nerofe+ldDox treatment increased IL-2 and suppressed IL-10 in PANC-1 cells, reversing the immunosuppressive cytokine profile. Patient biopsies confirmed these effects, showing higher IL-2, lower IL-10, and increased infiltration of NK cells, CD8^+^ cytotoxic T lymphocytes, and CD4^+^ helper T cells. KRAS protein levels were reduced in post-treatment biopsies. Mechanistically, Nerofe+ldDox elevated total c-Jun protein but reduced phosphorylation at Ser63 and Ser73. Co-immunoprecipitation showed that c-Jun was bound to nuclear ST2 under basal conditions; this complex was disrupted within 3 h of treatment, releasing c-Jun to activate IL-2 and miR-217 transcription before re-forming after 24 h. This transient release corresponds to the early induction of IL-2 and later reduction in KRAS levels.

Conclusions: Nerofe+ldDox reprograms the immune microenvironment of mtKRAS tumors by releasing c-Jun from inhibitory nuclear ST2, enabling expression of IL-2 and miR-217. This “nuclear immunomodulation” promotes immune cell infiltration and downregulates KRAS expression, highlighting Nerofe+ldDox as a promising therapeutic approach for mtKRAS-driven cancers.

## INTRODUCTION

KRAS mutations are among the most frequent oncogenic alterations in human cancers, particularly in pancreatic, colorectal, and lung adenocarcinomas [1, 2]. These mutations drive constitutive activation of downstream signaling pathways that promote tumor growth, survival, and immune evasion. Despite decades of research, direct pharmacological targeting of mutant KRAS (mtKRAS) has proven challenging, and only a limited subset of mutations can currently be addressed by targeted inhibitors [3].

A major obstacle in treating mtKRAS-driven tumors is their profoundly immunosuppressive microenvironment. Tumor cells secrete inhibitory cytokines such as interleukin-10 (IL-10) and transforming growth factor-β2 (TGF-β2), which prevent immune effector cells from infiltrating and attacking the tumor [4, 5]. As a result, mtKRAS tumors often show poor responses to conventional immunotherapies, including immune checkpoint inhibitors [6]. Strategies capable of reversing this immune exclusion and reprogramming the tumor microenvironment are therefore urgently needed.

Nerofe (dTCApFs) is a synthetic 14-amino-acid derivative of the Tumor Cell Apoptosis Factor peptide, originally identified by bioinformatics screening [7]. Preclinical studies demonstrated that Nerofe enters cells through the T1/ST2 receptor and induces apoptosis via disruption of Golgi function, induction of endoplasmic reticulum (ER) stress, and suppression of ER stress repair mechanisms [8]. Importantly, the ST2 receptor is sparsely expressed in most normal tissues but is strongly overexpressed in mtKRAS tumors, suggesting a therapeutic window [9]. A Phase I clinical trial in patients with advanced solid tumors established Nerofe as safe and potentially efficacious, and retrospective analyses showed better responses in patients whose tumors expressed high ST2 levels [10].

Nerofe induces surface exposure of calreticulin (CRT), an ‘eat-me’ signal for NK cells [11]. Moreover, combining Nerofe with certain chemotherapeutic agents may enhance these effects. Doxorubicin (DOX), a widely used anthracycline, is known to inhibit the unfolded protein response and at low doses can promote adaptive immune activation [12, 13]. Together, these findings support the rationale for combining Nerofe with low-dose doxorubicin (ldDox) to achieve synergistic anticancer activity. Indeed, in our previous study, Nerofe combined with doxorubicin converted mtKRAS tumors from an immunosuppressive to an immunostimulatory state with enhanced immune infiltration [14].

In this study, we investigated the molecular mechanism by which Nerofe+ldDox reprograms the immune behavior of mtKRAS tumors. Using PANC-1 pancreatic adenocarcinoma cells and biopsies from mtKRAS patients treated with Nerofe+ldDox, we focused on the nuclear receptor ST2 and its interaction with the transcription factor c-Jun. Our findings reveal a previously unrecognized mechanism of nuclear immunomodulation, whereby Nerofe+ldDox disrupts the inhibitory ST2–c-Jun complex, leading to activation of immune-stimulatory genes, infiltration of NK and T cells, and downregulation of KRAS expression. These insights highlight Nerofe+ldDox as a promising therapeutic approach for mtKRAS-driven cancers.

## RESULTS

### Nerofe+ldDox modulates cytokine expression in PANC-1 Cells

Treatment of PANC-1 cells with Nerofe in combination with low-dose doxorubicin (ldDox) produced opposite effects on IL-2 and IL-10 expression. Short-term exposure (3 h) induced IL-2 to 500% ±5% of control, demonstrating a strong early activation of this immune-stimulatory cytokine. In contrast, prolonged exposure (24 h) markedly suppressed the immunosuppressive cytokine IL-10, reducing its expression to 35% ±5% of control.

IL-10 and TGF-β2 were selected for analysis because they represent the two major immunosuppressive cytokines responsible for immune exclusion in mtKRAS tumors. These cytokines are central to maintaining the suppressive tumor microenvironment and are well-established drivers of T-cell and NK-cell exclusion. Therefore, they were prioritized in this study as the most biologically relevant markers of immunosuppression.

We also evaluated TGF-β2 expression in PANC-1 cells under the same treatment conditions; however, TGF-β2 levels remained unchanged across all time points.

This cytokine switch indicates that the combination treatment can reprogram the immune profile of mtKRAS tumor cells toward an immune-permissive state.

### Nerofe+ldDox alters the tumor microenvironment in patients with mtKRAS tumors

Patients with mtKRAS tumors received weekly Nerofe+ldDox for 7 weeks. Biopsies collected at screening and post-treatment were analyzed by IHC for IL-2, IL-10, CD56, CD8, CD4, and KRAS.

Tumor types: pt117 – head and neck squamous cell carcinoma, pt121 – rectal adenocarcinoma, pt122 – pancreatic adenocarcinoma.

**Figure 1:**
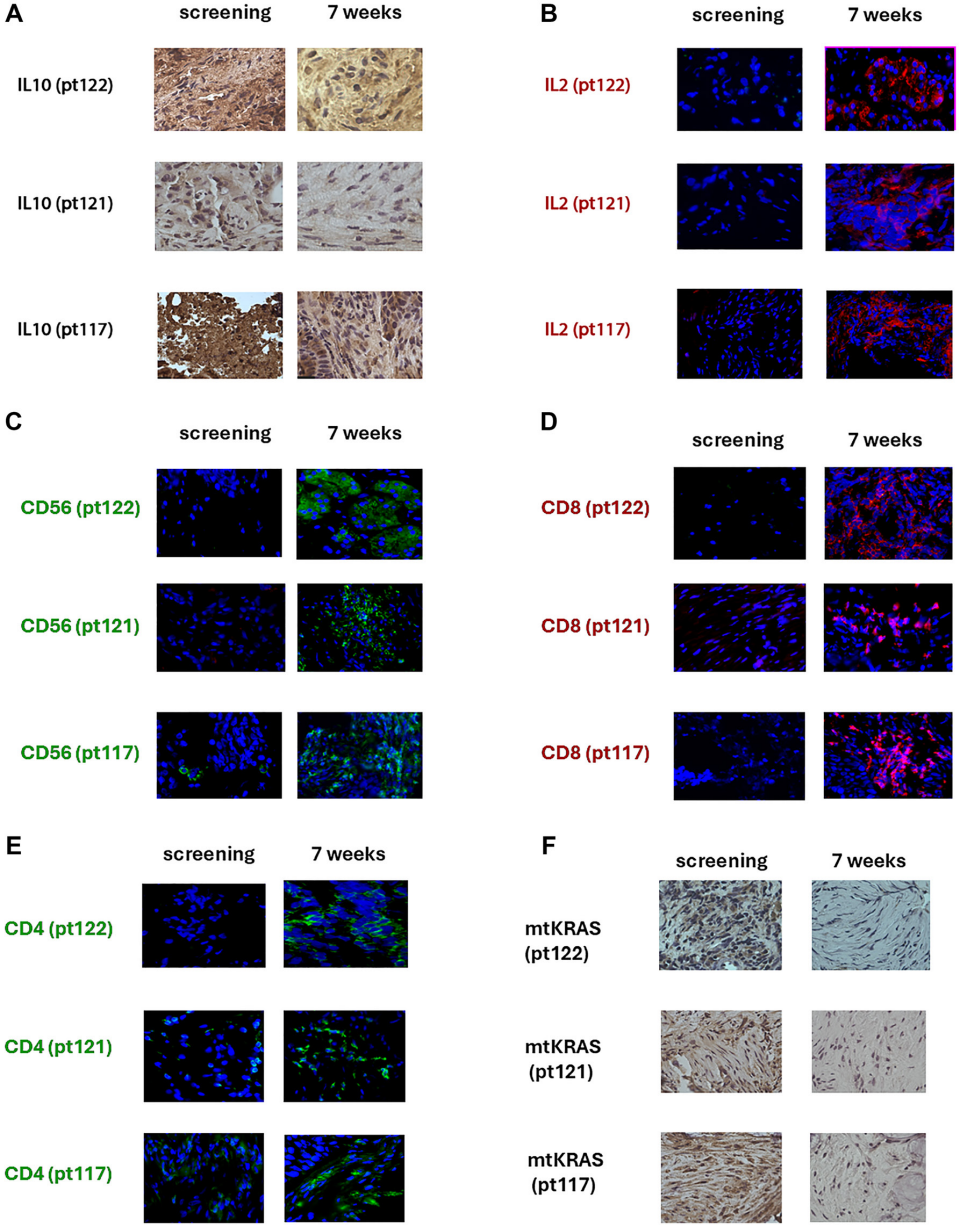
Patients with mtKRAS tumors received weekly Nerofe+ldDox for 7 weeks. Biopsies collected at screening and post-treatment were analyzed by IHC for IL-2, IL-10, CD56, CD8, CD4, and KRAS. Tumor types: pt117 – head and neck squamous cell carcinoma, pt121 – rectal adenocarcinoma, pt122 – pancreatic adenocarcinoma. Representative IHC images from all three patients are shown (panels A–F). (**A**) IL-10: All patients showed a marked reduction in IL-10 staining following treatment, indicating suppression of the immunosuppressive tumor milieu. (**B**) IL-2: Post-treatment biopsies demonstrated increased IL-2 expression in all three patients, consistent with activation of pro-immune signaling. (**C**) CD56: Increased infiltration of CD56^+^ NK cells was observed in post-treatment tissue. (**D**) CD8: Post-treatment biopsies revealed enhanced infiltration of CD8^+^ cytotoxic T lymphocytes. (**E**) CD4: Increased CD4^+^ T-cell presence was observed following treatment. (**F**) KRAS: KRAS staining intensity was reduced post-treatment across all three patients. This reduction reflects decreased KRAS protein expression rather than altered protein localization. TGF-β2 staining was also examined in patient biopsies, but no detectable changes in TGF-β2 expression were observed following treatment. All three patients carried activating KRAS mutations: pt117 (KRAS G12D), pt121 (KRAS G13D), and pt122 (KRAS G12D).

Representative IHC images from all three patients are shown in Figure 1 (panels A–F).

- IL-10: All patients showed a marked reduction in IL-10 staining following treatment, indicating suppression of the immunosuppressive tumor milieu.
- IL-2: Post-treatment biopsies demonstrated increased IL-2 expression in all three patients, consistent with activation of pro-immune signaling.
- CD56: Increased infiltration of CD56^+^ NK cells was observed in post-treatment tissue.
- CD8: Post-treatment biopsies revealed enhanced infiltration of CD8^+^ cytotoxic T lymphocytes.
- CD4: Increased CD4^+^ T-cell presence was observed following treatment.
- KRAS: KRAS staining intensity was reduced post-treatment across all three patients. This reduction reflects decreased KRAS protein expression rather than altered protein localization.

TGF-β2 staining was also examined in patient biopsies, but no detectable changes in TGF-β2 expression were observed following treatment.

All three patients carried activating KRAS mutations: pt117 (KRAS G12D), pt121 (KRAS G13D), and pt122 (KRAS G12D).

### IL-2 induction by Nerofe+ldDox depends on AP-1 activity

To determine whether IL-2 induction was mediated by AP-1, PANC-1 cells were treated for 3 h with Nerofe+ldDox in the presence or absence of the AP-1 inhibitor SR11302. Western blot quantification was performed by densitometry and normalized to actin. Data are presented as mean ± SD of three independent experiments. Treatment with Nerofe+ldDox induced IL-2, and this induction was completely blocked by AP-1 inhibition, demonstrating that IL-2 upregulation is AP-1-dependent.

### Effect of Nerofe+ldDox on c-Jun induction and phosphorylation in PANC-1 cells

PANC-1 cells were treated for the indicated times with Nerofe+ldDox. Western blot quantification was performed by densitometry and normalized to actin. Data are presented as mean ± SD of at least three independent experiments. Treatment increased total c-Jun protein to 238% ±5% of control at 3 h, reflecting early promoter activation. In contrast, phosphorylation at Ser63 decreased to 64% ±5% of control, and phosphorylation at Ser73 decreased to 21% ±5% of control overnight. These dynamic changes indicate an early activation phase followed by reduced c-Jun activity due to progressive loss of phosphorylation.

### Effect of Nerofe+ldDox on nuclear ST2 binding to c-Jun

- Nuclear extracts from PANC-1 cells were immunoprecipitated (IP) with an anti–c-Jun antibody and analyzed by Western blotting using anti-ST2 antibody. In untreated cells, nuclear ST2 was bound to immunoprecipitated c-Jun. This interaction was strongly reduced after 3 h of Nerofe+ldDox treatment.
- By 24 h, the ST2–c-Jun interaction re-formed, indicating a reversible and dynamic inhibitory mechanism.

These findings were validated with two independent anti-ST2 antibodies, confirming the specificity of the nuclear ST2–c-Jun interaction.

## DISCUSSION

In our earlier work, we reported that Nerofe induces severe ER stress [8]. Given these immune changes, we next investigated which intracellular processes enable IL-2 induction and KRAS reduction. In this study, we demonstrate that Nerofe combined with low-dose doxorubicin (ldDox) induces IL-2 expression in human pancreatic adenocarcinoma cells (PANC-1) through activation of the AP-1 transcription factor. Notably, the combination alters c-Jun activity by releasing it from its interaction with the nuclear receptor ST2, which likely acts as an inhibitory regulator of c-Jun function.

**Figure 2:**
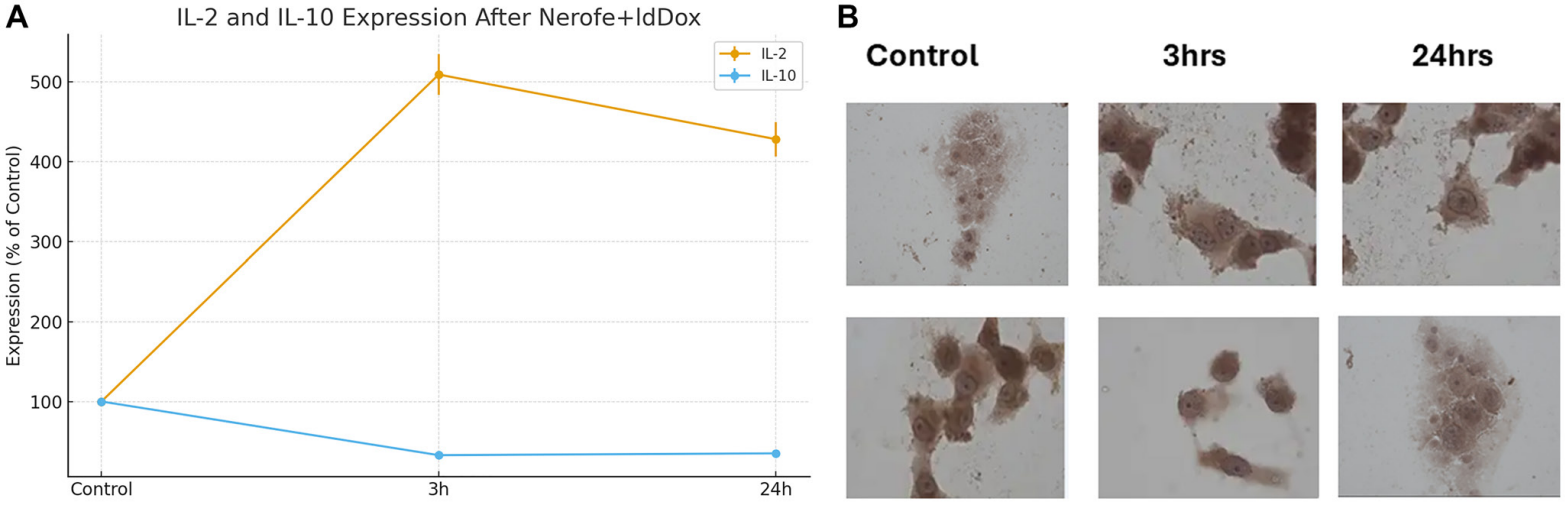
Treatment of PANC-1 cells with Nerofe in combination with low-dose doxorubicin (ldDox) produced opposite effects on IL-2 and IL-10 expression. (**A**, **B**) Short-term exposure (3 h) induced IL-2 to 500% ± 5% of control, demonstrating a strong early activation of this immune- stimulatory cytokine. In contrast, prolonged exposure (24 h) markedly suppressed the immunosuppressive cytokine IL-10, reducing its expression to 35% ± 5% of control. IL-10 and TGF-β2 were selected for analysis because they represent the two major immunosuppressive cytokines responsible for immune exclusion in mtKRAS tumors. These cytokines are central to maintaining the suppressive tumor microenvironment and are well- established drivers of T-cell and NK-cell exclusion. Therefore, they were prioritized in this study as the most biologically relevant markers of immunosuppression. TGF-β2 expression in PANC-1 cells remained unchanged under all treatment conditions. This cytokine switch indicates that the combination treatment reprograms the immune profile of mtKRAS tumor cells toward an immune-permissive state.

Our *in vitro* experiments showed that Nerofe+ldDox increased IL-2 expression while simultaneously reducing IL-10 in PANC-1 cells (Figure 2). Because PANC-1 cells harbor mtKRAS mutations, this shift is highly meaningful: IL-2 is an immune-stimulating cytokine, whereas IL-10 is strongly immunosuppressive. Consistent with these findings, our previous study in an mtKRAS colorectal cancer mouse model demonstrated that Nerofe+ldDox increased infiltration of NK cells and M1 monocytes.

In our ongoing clinical trial (NCT05661201), patients with mtKRAS tumors received weekly Nerofe (288 mg/m²) and ldDox (8 mg/m²). Tumor biopsies collected before treatment and after seven weeks were analyzed by IHC. As shown in Figure 1, patients 117, 121, and 122 exhibited clear remodeling of their tumor microenvironments, with increased IL-2 and decreased IL-10 expression, mirroring our *in vitro* and murine results. Importantly, NK cells, CD8^+^ cytotoxic T lymphocytes, and CD4^+^ helper T cells were detected infiltrating the tumors, indicating a strong immune response initiated by the combination therapy.

Although additional KRAS-mutant cancer cell lines could be examined, the mechanistic conclusions of this study are strengthened by the inclusion of tumor biopsies from three mtKRAS-positive patients. These patient-derived samples demonstrate that the Nerofe+ldDox mechanism operates in human tumors, providing direct clinical relevance beyond what additional *in vitro* lines would offer. Since the goal of this work was to define a specific mechanistic pathway—namely release of c-Jun from nuclear ST2—rather than to perform broad screening across multiple lines, the combination of a well-characterized KRAS-mutant model (PANC-1) with three independent patient tumors provides strong biological validation.

Another key observation was that Nerofe+ldDox reduced mtKRAS protein expression in patients 117, 121, and 122. We propose that this effect may be mediated through induction of miR-217, which has been reported to suppress KRAS expression.

It is important to clarify the mechanistic role of low-dose doxorubicin (ldDox) in this combination therapy. In this study, ldDox was not used as a cytotoxic chemotherapeutic agent but rather as a mechanistic enhancer of Nerofe activity. Specifically, ldDox functions as an inhibitor of the IRE-1 arm of the unfolded protein response (UPR), thereby impairing the cellular ability to resolve Nerofe-induced ER stress. We previously demonstrated that Nerofe induces severe ER stress leading to apoptosis, and that doxorubicin synergistically enhances this effect by blocking ER-stress recovery (Ohana et al., 2017; reference 24). Consistent with this mechanism, ldDox alone does not induce IL-2 expression, does not suppress IL-10, does not induce miR-217, and does not reduce KRAS protein levels—effects that we observed only when ldDox was combined with Nerofe. Additionally, ldDox is used here at immune-modulatory concentrations known to promote adaptive immune activation without exerting direct cytotoxicity. Thus, the contribution of ldDox in our system is twofold: (i) to potentiate Nerofe-driven ER-stress–mediated signaling, and (ii) to support activation of innate and adaptive anti-tumor immunity, consistent with previous literature on low-dose anthracycline immune priming.

**Figure 3:**
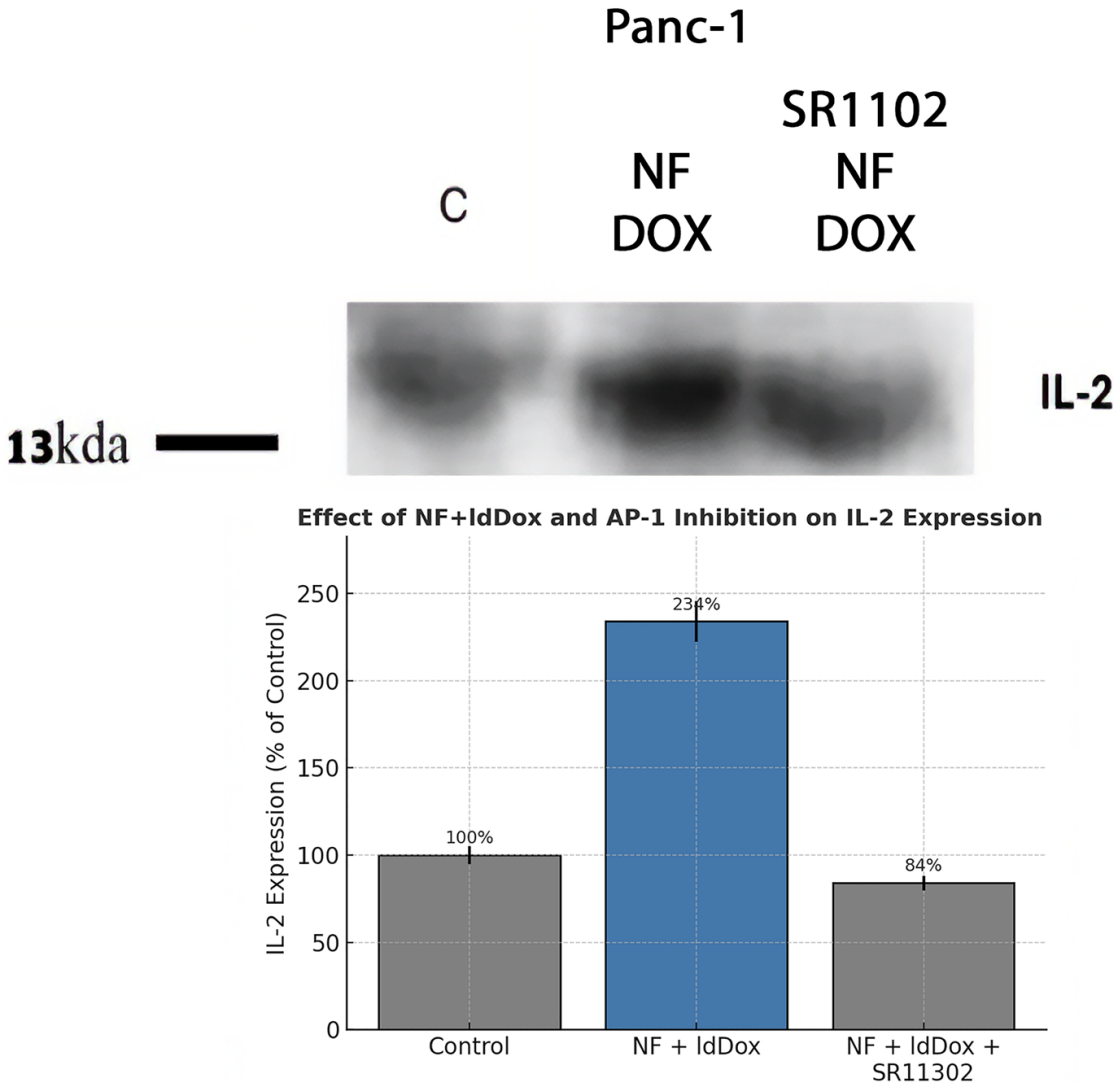
To determine whether IL-2 induction was mediated by AP-1, PANC-1 cells were treated for 3 h with Nerofe+ldDox in the presence or absence of the AP-1 inhibitor SR11302. Western blot quantification was performed by densitometry and normalized to actin. Data are presented as mean ± SD of three independent experiments. Treatment with Nerofe+ldDox induced IL-2 expression, and this induction was completely blocked by AP-1 inhibition, demonstrating that IL-2 upregulation is AP-1-dependent.

**Figure 4:**
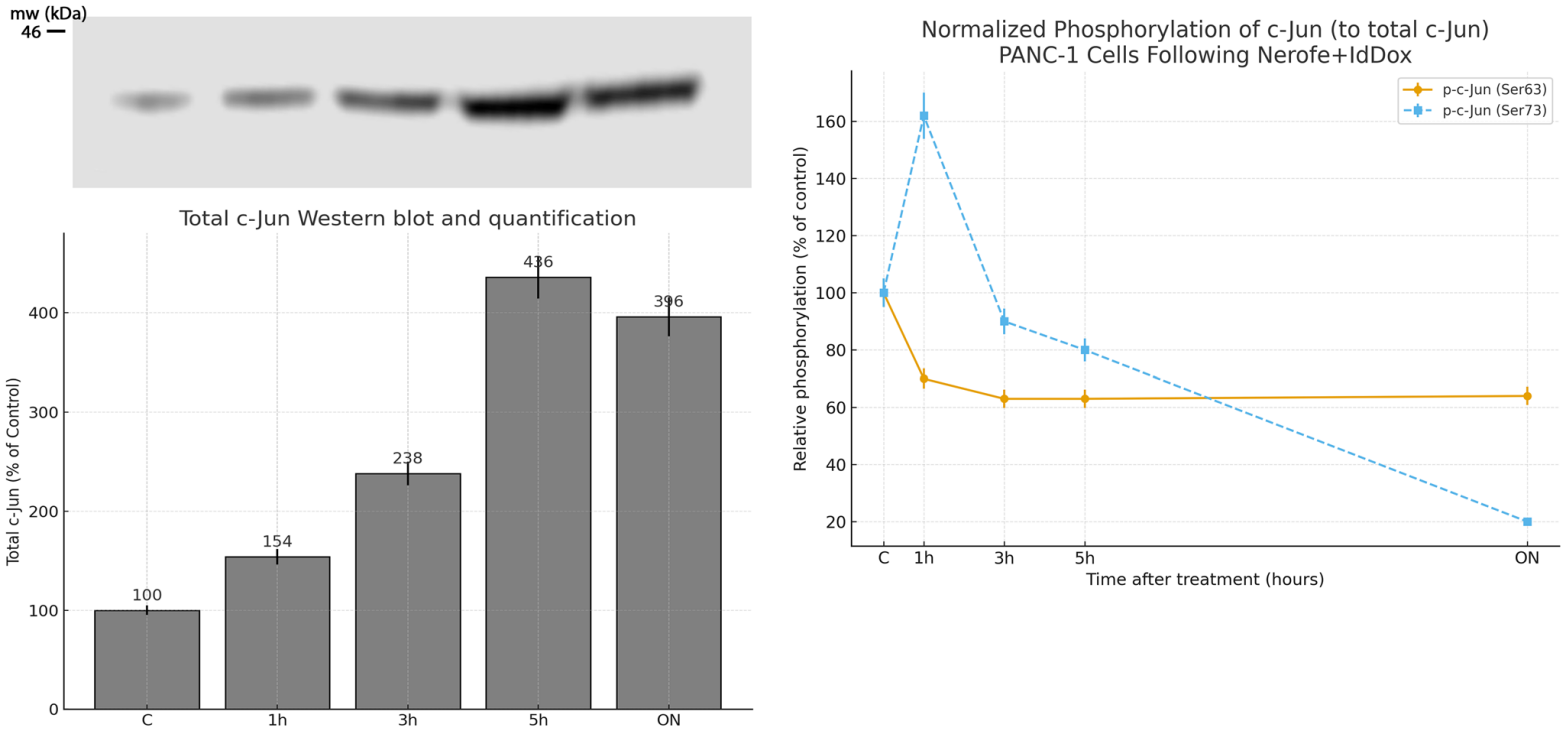
PANC-1 cells were treated for the indicated times with Nerofe+ldDox. Western blot quantification was performed by densitometry and normalized to actin. Data are presented as mean ± SD of at least three independent experiments. Treatment increased total c-Jun protein to 238% ± 5% of control at 3 h, reflecting early promoter activation. In contrast, phosphorylation at Ser63 decreased to 64% ± 5% of control, and phosphorylation at Ser73 decreased to 21% ± 5% of control overnight. These dynamic changes indicate an early activation phase followed by reduced c-Jun activity due to progressive loss of phosphorylation.

To confirm the role of c-Jun, we tested whether IL-2 induction by Nerofe+ldDox required AP-1 activity using the AP-1 inhibitor SR11302. As shown in Figure 3, the inhibitor blocked IL-2 induction, confirming that c-Jun mediates this effect. We then analyzed c-Jun protein levels and post-translational modifications. Treatment with Nerofe+ldDox increased total c-Jun protein (Figure 4), but phosphorylation at Ser63 and Ser73 decreased significantly (≈36% and ≈79% overnight, respectively), suggesting early c-Jun activation followed by a decline as phosphorylation wanes. This may explain why IL-2 expression increases within three hours of treatment, while IL-10 levels decline more gradually.

In contrast, TGF-β2 expression did not change significantly *in vitro* or in patient biopsies. Although TGF-β2 is a major immunosuppressive cytokine in mtKRAS tumors, our data indicate that the Nerofe+ldDox combination preferentially modulates IL-2 and IL-10, while leaving TGF-β2 unaffected.

The early rise in total c-Jun protein following Nerofe+ldDox exposure is consistent with transcriptional activation of the c-Jun promoter, which requires initial phosphorylation events downstream of AP-1 regulatory signaling. This early phosphorylation facilitates rapid c-Jun accumulation and enables activation of IL-2 transcription. At later time points, the marked decline in phospho-Ser63 and phospho-Ser73 indicates a loss of activating phosphorylation, resulting in reduced c-Jun transcriptional activity after the initial induction phase. This temporal pattern supports a model in which Nerofe+ldDox triggers an early, phosphorylation-dependent activation of c-Jun, followed by a regulated decrease in phospho-c-Jun that attenuates activity over time.

**Figure 5:**
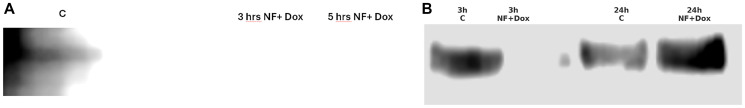
(**A**) Nuclear extracts from PANC-1 cells were immunoprecipitated with an anti-c-Jun antibody and analyzed by Western blotting using anti-ST2 antibody. In untreated cells, nuclear ST2 was bound to immunoprecipitated c-Jun. This interaction was strongly reduced after 3 h of Nerofe+ldDox treatment. (**B**) By 24 h, the ST2–c-Jun interaction re-formed, indicating a reversible and dynamic inhibitory mechanism. These findings were validated with two independent anti-ST2 antibodies, confirming the specificity of the nuclear ST2–c-Jun interaction.

A key question was how Nerofe+ldDox regulates c-Jun activity. Since both c-Jun and ST2 localize to the nucleus, we tested whether they physically interact. Co-immunoprecipitation experiments revealed that c-Jun and the inhibitory nuclear isoform of ST2 form a complex in untreated PANC-1 cells (Figure 5A, 5B). This suggests that ST2 negatively regulates c-Jun by direct interaction. Following Nerofe+ldDox treatment, this complex was disrupted: within three hours, c-Jun was largely released from ST2, allowing it to activate transcription of target genes. By 24 h, the complex re-formed, suggesting a dynamic regulatory mechanism. These findings were validated with the listed ST2 antibody (Figure 5). Notably, c-Fos was not found to be part of this complex (data not shown).

Taken together, these findings describe a novel immunotherapeutic mechanism based on nuclear regulation of cytokine gene transcription. This study was designed specifically to evaluate immune remodeling mechanisms rather than apoptosis. For this reason, classical apoptotic markers (cleaved caspase-3, Annexin V, PARP cleavage) were not included in the current experimental panel. The apoptotic activity of Nerofe has already been established in our previous studies, where Nerofe induced strong ER-stress–mediated apoptosis through Golgi disruption. Here, we focused solely on the immune regulatory arm of the response, including IL-2, IL-10, and c-Jun/ST2 signaling. The inhibitory nuclear form of ST2 binds c-Jun and suppresses its activity. Upon treatment with Nerofe+ldDox, c-Jun is released from this inhibitory complex, enabling activation of IL-2 and miR-217. Increased IL-2 expression shifts the immune response toward tumor attack, allowing NK cells, CD8+ T cells, and CD4+ T cells to infiltrate the tumor microenvironment, while miR-217 downregulates mtKRAS expression.

**Figure 6:**
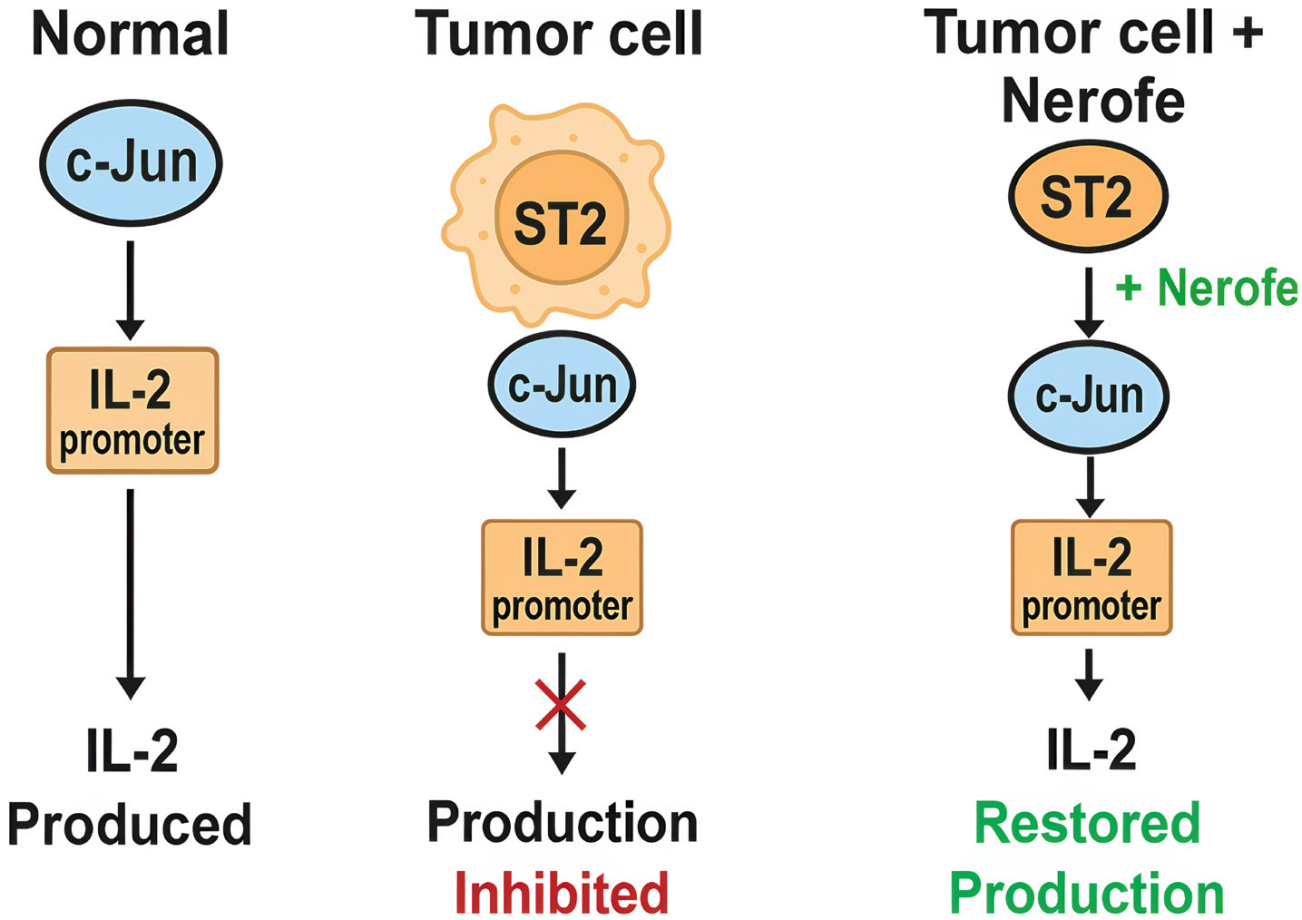
Proposed model of nuclear immunomodulation. In tumor cells, nuclear ST2 binds and inhibits c-Jun, preventing activation of the IL-2 promoter. Treatment with Nerofe+ldDox disrupts this inhibitory complex, releasing c-Jun and enabling induction of IL-2 and miR- 217, leading to immune activation and reduction of KRAS expression.

As illustrated in Figure 6, in tumor cells nuclear ST2 binds and inhibits c-Jun, preventing IL-2 production. Treatment with Nerofe+ldDox disrupts the ST2–c-Jun complex, restoring IL-2 expression and activating immune-stimulatory pathways.

## MATERIALS AND METHODS

### Cell culture and treatments

Human pancreatic adenocarcinoma PANC-1 cells (ATCC^®^ CRL-1469^™^), harboring a KRAS mutation, were maintained in RPMI-1640 medium (Gibco, Thermo Fisher Scientific) supplemented with 2% fetal bovine serum (FBS) at 37°C in a humidified atmosphere with 5% CO_2_. Cells were seeded in 175 cm² flasks and grown to 70–80% confluence. For treatments, media were replaced with RPMI-1640 containing 2% FBS and 5% mannitol. Cells were exposed for 3–24 h to Nerofe (dTCApFs; Immune System Key Ltd., Jerusalem, Israel) at 15 or 50 μg/mL and low-dose doxorubicin (DOX; Sigma-Aldrich) at 0.5 μM, alone or in combination.

A doxorubicin-only control group was not included because low-dose DOX (0.5 μM) has been repeatedly shown—both in our 2017 and 2023 publications—not to induce IL-2, IL-10, miR-217, KRAS suppression, or ER-stress–related apoptosis. Its function in this study is restricted to inhibition of the IRE1 pathway to potentiate the ER-stress induced by Nerofe, not to exert direct cytotoxicity. Therefore, DOX alone is not expected to produce measurable biological effects under these conditions. All comparisons in this study therefore reference either untreated controls or Nerofe+ldDox.

All experiments were performed in at least three independent biological replicates, with comparable results. Each assay also included technical replicates as appropriate.

### Nuclear extraction

Nuclear proteins were isolated using the Abcam Nuclear Extraction Kit (ab113474) following the manufacturer’s instructions. Briefly, cells were harvested, washed in PBS, and lysed in Pre-Extraction Buffer containing dithiothreitol (DTT) and protease inhibitor cocktail. Cytoplasmic fractions were removed by centrifugation. Nuclear pellets were lysed in Extraction Buffer with DTT and protease inhibitors, incubated on ice, vortexed intermittently, and centrifuged. Nuclear supernatants were immediately used for immunoprecipitation (IP) or Western blot (WB) analysis.

### Immunoprecipitation and western blotting

Nuclear extracts (100–500 μg protein) were incubated with 2 μg anti–c-Jun antibody (Rabbit mAb 60A8, Santa Cruz Biotechnology, sc-1694) for 1 h at 4°C with rotation. Protein A/G PLUS-Agarose beads (Santa Cruz Biotechnology, sc-2003) were added and incubated overnight. Beads were washed four times with RIPA buffer, and bound proteins were eluted in sample buffer, denatured, and subjected to SDS-PAGE (4–20% gradient gels). Proteins were transferred to nitrocellulose membranes and probed with rabbit anti-ST2 antibody (Abcam, ab233433; 1:1000), followed by HRP-conjugated secondary antibody. Detection was performed by enhanced chemiluminescence (ECL).

All Western blot quantifications were normalized to actin as a housekeeping control. For Figures 3 and 5, equal numbers of cells were used for nuclear extraction and immunoprecipitation to ensure that band intensities reflect biological differences rather than cell loading variability.

### Antibodies for western blotting and immunohistochemistry

- IL-2: goat anti-human IL-2 (R&D Systems, AF-502-NA; 1:100).
- IL-10: rabbit anti-human IL-10 (Abcam, ab134742; 1:100).
- TGF-β2: rabbit anti-human TGF-β2 (Abcam, ab80059; 1:100).
- c-Jun (IP antibody): rabbit mAb clone 60A8 (Santa Cruz Biotechnology, sc-1694; 2 μg/IP).
- ST2 (WB antibody after IP): rabbit anti-human ST2 (Abcam, ab233433; 1:1000).
- ST2 (confirmation antibody): affinity-purified ST2 antibody (homemade). Used for verification of IP-WB results (data not shown).
- CD56: rabbit monoclonal anti-human CD56/N-CAM (Cell Signaling Technology, #3576; 1:100).
- CD8: rabbit monoclonal anti-human CD8α (Abcam, ab203035; 1:100).
- CD4: rabbit polyclonal anti-human CD4 (Santa Cruz Biotechnology, sc-12730; 1:100).
- KRAS: mouse monoclonal anti-human KRAS, clone 9.13 (LSBio, LS-C354935; 1:100).

### Cytokine detection

Expression of IL-2 and IL-10 in PANC-1 cells was analyzed by Western blotting and by immunocytochemistry using the antibodies described above.

### Patient biopsies and immunohistochemistry

Patients with mtKRAS-positive tumors were enrolled in a Phase Ib/IIa clinical trial (NCT05661201). Patient recruitment for this trial took place during 2024–2025, and the study is ongoing. Subjects received weekly intravenous Nerofe plus low-dose doxorubicin (ldDox) for seven weeks. Patients were enrolled in different dosing cohorts of the ongoing clinical trial (NCT05661201). Accordingly, pt117 received 192 mg/m² Nerofe, while pt121 and pt122 received 288 mg/m² Nerofe. All patients received the same ldDox dose (8 mg/m²). Despite the cohort-based dosing differences, the immunologic and KRAS-related treatment effects were consistent across all patients.

Tumor biopsies were obtained at baseline and after treatment, fixed in formalin, paraffin-embedded, and analyzed by immunohistochemistry (IHC). Sections were stained with antibodies against IL-2, IL-10, CD56, CD8, CD4, and KRAS, and evaluated for changes in protein expression and immune cell infiltration.

The three mtKRAS-positive patients included in this study carried the following KRAS mutations: pt117 – G12D, pt121 – G13D, and pt122 – G12D. These patients were treated in two dose cohorts of the ongoing clinical trial: pt117 received 192 mg/m² Nerofe, while pt121 and pt122 each received 288 mg/m² Nerofe. All patients received low-dose doxorubicin (8 mg/m²) once weekly. Tumor biopsies were obtained before treatment (screening) and after 7 weeks.

### Ethical approval

All patient studies were performed in accordance with the Declaration of Helsinki. The protocol was approved by the Ethics Committee of Georgetown University, Washington, DC (protocol code NCT05661201). Written informed consent was obtained from all participants.

### Use of generative AI

Generative artificial intelligence (AI) was used only for language polishing. No AI was used for data generation, analysis, or interpretation. The authors reviewed and edited all AI-generated content and take full responsibility for the final manuscript.

## CONCLUSIONS

In this study, we demonstrate that the combination of Nerofe and low-dose doxorubicin (ldDox) reprograms the immune microenvironment of mtKRAS-driven tumors. In PANC-1 cells, the treatment induced IL-2 while suppressing IL-10, thereby shifting the cytokine profile from immunosuppressive to immune-stimulatory. These effects were confirmed in patient biopsies, which showed increased infiltration of NK cells, CD8^+^ cytotoxic T cells, and CD4^+^ helper T cells, together with reduced KRAS protein expression.

Mechanistically, we identified a novel regulatory process, which we term nuclear immunomodulation: under basal conditions, the transcription factor c-Jun is sequestered by the nuclear ST2 receptor, limiting its activity. Treatment with Nerofe+ldDox disrupted this inhibitory interaction, as validated with two independent ST2 antibodies (Figure 5), releasing c-Jun to activate transcription of IL-2 and miR-217, and downregulate KRAS expression.

## Abbreviations

mtKRAS: mutant KRAS
ldDox: low-dose doxorubicin
ER: endoplasmic reticulum
NK: natural killer
IHC: immunohistochemistry
IP: immunoprecipitation
WB: western blot

## References

[1] Prior IA , Hood FE , Hartley JL . The Frequency of Ras Mutations in Cancer. Cancer Res. 2020; 80:2969–74. 10.1158/0008-5472.CAN-19-3682. 32209560 PMC7367715

[2] Simanshu DK , Nissley DV , McCormick F . RAS Proteins and Their Regulators in Human Disease. Cell. 2017; 170:17–33. 10.1016/j.cell.2017.06.009. 28666118 PMC5555610

[3] Canon J , Rex K , Saiki AY , Mohr C , Cooke K , Bagal D , Gaida K , Holt T , Knutson CG , Koppada N , Lanman BA , Werner J , Rapaport AS , et al. The clinical KRAS(G12C) inhibitor AMG 510 drives anti-tumour immunity. Nature. 2019; 575:217–23. 10.1038/s41586-019-1694-1. 31666701

[4] Ostrand-Rosenberg S , Sinha P . Myeloid-derived suppressor cells: linking inflammation and cancer. J Immunol. 2009; 182:4499–506. 10.4049/jimmunol.0802740. 19342621 PMC2810498

[5] Flavell RA , Sanjabi S , Wrzesinski SH , Licona-Limón P . The polarization of immune cells in the tumour environment by TGFbeta. Nat Rev Immunol. 2010; 10:554–67. 10.1038/nri2808. 20616810 PMC3885992

[6] Ribas A , Wolchok JD . Cancer immunotherapy using checkpoint blockade. Science. 2018; 359:1350–55. 10.1126/science.aar4060. 29567705 PMC7391259

[7] Sandler U , Devary O , Braitbard O , Ohana J , Kass G , Rubinstein AM , Friedman ZY , Devary Y . NEROFE--a novel human hormone-peptide with anti-cancer activity. J Exp Ther Oncol. 2010; 8:327–39. 21222365

[8] Ohana J , Sandler U , Kass G , Stemmer SM , Devary Y . dTCApFs, a derivative of a novel human hormone peptide, induces apoptosis in cancer cells through a mechanism involving loss of Golgi function. Mol Clin Oncol. 2017; 7:991–99. 10.3892/mco.2017.1453. 29285362 PMC5740848

[9] Tago K , Ohta S , Kashiwada M , Funakoshi-Tago M , Matsugi J , Tominaga SI , Yanagisawa K . ST2 gene products critically contribute to cellular transformation caused by an oncogenic Ras mutant. Heliyon. 2017; 3:e00436. 10.1016/j.heliyon.2017.e00436. 29226265 PMC5714553

[10] Stemmer SM , Benjaminov O , Silverman MH , Sandler U , Purim O , Sender N , Meir C , Oren-Apoteker P , Ohana J , Devary Y . A phase I clinical trial of dTCApFs, a derivative of a novel human hormone peptide, for the treatment of advanced/metastatic solid tumors. Mol Clin Oncol. 2018; 8:22–29. 10.3892/mco.2017.1505. 29423221 PMC5772927

[11] Obeid M , Tesniere A , Ghiringhelli F , Fimia GM , Apetoh L , Perfettini JL , Castedo M , Mignot G , Panaretakis T , Casares N , Métivier D , Larochette N , van Endert P , et al. Calreticulin exposure dictates the immunogenicity of cancer cell death. Nat Med. 2007; 13:54–61. 10.1038/nm1523. 17187072

[12] Jiang D , Lynch C , Medeiros BC , Liedtke M , Bam R , Tam AB , Yang Z , Alagappan M , Abidi P , Le QT , Giaccia AJ , Denko NC , Niwa M , Koong AC . Identification of Doxorubicin as an Inhibitor of the IRE1α-XBP1 Axis of the Unfolded Protein Response. Sci Rep. 2016; 6:33353. 10.1038/srep33353. 27634301 PMC5025885

[13] Voorwerk L , Slagter M , Horlings HM , Sikorska K , van de Vijver KK , de Maaker M , Nederlof I , Kluin RJC , Warren S , Ong S , Wiersma TG , Russell NS , Lalezari F , et al. Immune induction strategies in metastatic triple-negative breast cancer to enhance the sensitivity to PD-1 blockade: the TONIC trial. Nat Med. 2019; 25:920–28. 10.1038/s41591-019-0432-4. 31086347

[14] Ohana J , Sandler U , Devary O , Devary Y . Transformation of immunosuppressive mtKRAS tumors into immunostimulatory tumors by Nerofe and Doxorubicin. Oncotarget. 2023; 14:688–99. 10.18632/oncotarget.28467. 37395796 PMC10317071

[15] Zhao WG , Yu SN , Lu ZH , Ma YH , Gu YM , Chen J . The miR-217 microRNA functions as a potential tumor suppressor in pancreatic ductal adenocarcinoma by targeting KRAS. Carcinogenesis. 2010; 31:1726–33. 10.1093/carcin/bgq160. 20675343

[16] Xiao Y , Deng T , Su C , Shang Z . MicroRNA 217 inhibits cell proliferation and enhances chemosensitivity to doxorubicin in acute myeloid leukemia by targeting KRAS. Oncol Lett. 2017; 13:4986–94. 10.3892/ol.2017.6076. 28599501 PMC5453027

[17] Xu X , Jiang Y , Wang J , Shi W , Liu Q , Liu Y . MicroRNA-217 regulates KRAS and inhibits tumorigenesis in acute myeloid leukemia. Mol Cancer. 2017; 16:158. 29025423

