# Correction: Nerofe+ldDox releases c-Jun from nuclear ST2 to reprogram the immune microenvironment in mtKRAS tumors

**DOI:** 10.18632/oncotarget.28859

**Published:** 2026-04-24

**Authors:** Joel Ohana, Uziel Sandler, Benjamin A. Weinberg, Stephen Liu, Yoram Devary

**Affiliations:** ^1^Immune System Key (ISK) Ltd., Jerusalem 9746009, Israel; ^2^Department of Bio-Informatics, Lev Academic Center (JCT), Jerusalem 91160, Israel; ^3^Ruesch Center for the Cure of Gastrointestinal Cancers, Lombardi Comprehensive Cancer Center, Georgetown University, Washington, DC 20007, USA

**This article has been corrected:** The authors of the article have provided a correction for the “Conflicts of Interest” section. The corrected text is shown below.

